# Mechanism of Xiao-ai-fei Honey Ointment, a Traditional Uyghur Multi-Ingredient Medicinal Preparation, Against Cervical Cancer Based on Network Pharmacology and In Vitro Evaluation of Anti-Cancer Activity

**DOI:** 10.3390/ph19050686

**Published:** 2026-04-27

**Authors:** Xiariwana Abasi, Di Liang, Remila Rezhake, Gulixian Tuerxun, Qian Zhuo, Xian Ju, Hongyu Su, Jing Yang, Guzhalinuer Abulizi

**Affiliations:** 1 The Third Clinical Medical College, Xinjiang Medical University, Urumqi 830000, China; 2 School of Forensic Medicine, Shanxi Medical University, Jinzhong 030600, China; 3 West China School of Basic Medical Sciences and Forensic Medicine, Sichuan University, Chengdu 610041, China; 4 Xinjiang Key Laboratory of Oncology, Affiliated Cancer Hospital of Xinjiang Medical University, Urumqi 830000, China

**Keywords:** cervical cancer, network pharmacology, single-cell sequencing, molecular docking

## Abstract

**Background/Objectives:** Cervical cancer, primarily driven by persistent high-risk HPV infection, remains a major global health issue. Xiao-ai-fei honey ointment, a traditional Uyghur multi-ingredient preparation, has shown clinical promise in cancer treatment, but its mechanisms against cervical cancer are not fully understood. This study aimed to investigate the potential molecular mechanisms of ethanolic extract of Xiao-ai-fei honey ointment (XAFHO) in cervical cancer using network pharmacology, single-cell RNA sequencing, and experimental validation. **Methods:** Differentially expressed genes (DEGs) in cervical cancer were identified from TCGA database. Active components and corresponding targets of XAFHO were retrieved from the TCMSP database, and disease targets were obtained from GeneCard, OMIM, and the TTD. Intersection targets were subjected to multivariate Cox and LASSO regression to construct a prognostic model. Immune infiltration, TMB, and MSI were compared between risk groups. Single-cell RNA-seq data were analyzed to determine cellular origins and inter-cellular communication. In vitro assays were performed on HeLa and SiHa cells to assess the anti-cancer activity of XAFHO. Molecular docking evaluated binding affinities between active compounds and core targets. The expression and functional roles of *FASN* and *SPP1* were further validated by RT-qPCR, Western blotting, and siRNA transfection. **Results:** Sixty-three potential XAFHO targets were identified, and an 11-gene prognostic model was established, effectively stratifying patients into high- and low-risk groups with significantly different overall survival (AUC > 0.7). The high-risk group exhibited an immunosuppressive microenvironment and higher TMB. Single-cell analysis revealed that *FASN* and *ACACA* were predominantly expressed in tumor cells, while *SPP1* was enriched in macrophages/monocytes. Tumor cells communicated with immune cells via the TGFB1–TGFβR1/R2 axis, promoting immune evasion. In vitro, XAFHO significantly inhibited proliferation, colony formation, migration, and invasion of cervical cancer cells. Molecular docking confirmed the strong binding of quercetin, kaempferol, and isorhamnetin to *FASN* and *SPP1* (binding energy < –6.0 kcal/mol). Functional validation indicated that upregulated *FASN* and *SPP1* contribute to malignant behaviors in cervical cancer cells. **Conclusions:** This study integrates network pharmacology with single-cell and experimental approaches to demonstrate that XAFHO exerts multi-target and multi-cell anti-cervical cancer effects, potentially by modulating lipid metabolism and immune-related pathways via *FASN* and *SPP1*. These findings provide a scientific basis for the therapeutic application of XAFHO in cervical cancer.

## 1. Introduction

Cervical cancer is the fourth most common malignancy among women worldwide, with its pathogenesis being closely associated with persistent human papillomavirus (HPV) infection [1,2]. High-risk HPV infection is widely recognized as the primary etiological factor for cervical cancer [3]. Early-stage cervical cancer is typically managed through surgical intervention, whereas advanced stages are commonly treated with a combination of chemotherapy and radiotherapy [4]. Although targeted therapies and immunotherapies have significantly improved patient survival, not all patients respond favorably to these treatments [5]. This underscores that tumor heterogeneity remains a critical obstacle in developing effective therapeutics to improve patient prognosis [6]. Consequently, there is an urgent need to develop novel agents to extend the survival of cervical cancer patients.

Xiao-ai-fei honey ointment is a traditional Uyghur medicinal formulation with a long history of use in China for cancer treatment and in clinical practice, and it is composed of five Uyghur medicinal components: *Bungarus multicinctus*, *Biba* (*Piperis Longi Fructus*), *Gaoliangjiang* (*Rhizoma Alpiniae Officinarum*), *Hujiao* (*Piper nigrum L*), and *Ganjiang* (*Zingiber officinale Roseco*) [7,8,9]. This formulation has been primarily utilized in the treatment of tumors, as well as inflammatory conditions, leakage syndromes, and refractory ulcers affecting the digestive and reproductive systems, including obstinate and malignant sores associated with qi stagnation [7].

*Zaocys dhumnades*, the dried body of the juvenile Chinese many-banded krait, is a traditional Chinese medicinal material. Chemical analyses have shown that it contains more than 20 elements [10] and has been clinically employed in the treatment of rheumatism, tetanus, and leprosy [11]. *Piper longum L*, which serves both as a traditional Chinese medicine and a food, is classified under the Piper genus of the Piperaceae family [12]. It possesses various pharmacological activities, including anti-inflammatory, antioxidant, and cardiovascular protective effects, primarily attributed to its rich content of amide alkaloids [13]. *Alpinia officinarum Hance*, derived from the dried roots and rhizomes of Alpinia officinarum, has been reported to exhibit antioxidant, antidiabetic, anti-inflammatory, and anticoagulant activities [14,15]. *Piper nigrum L* is derived from the flowering vines of the Piperaceae family [16]. This species has been extensively investigated for its diverse biological activities and the presence of bioactive phytochemical compounds. Current studies have revealed that *Piper nigrum L* exhibits varying degrees of inhibitory effects on a variety of cancers, including breast cancer and gastric cancer [17,18]. *Zingiber officinale Roscoe* is the dried rhizome of ginger family plants [19]. Its chemical constituents are primarily volatile and pungent compounds. Modern pharmacological studies have demonstrated that ginger possesses analgesic, anti-inflammatory, antibacterial, anti-tumor, and local circulation-improving properties [20]. Although these agents have demonstrated a certain degree of anti-tumor activity, their mechanisms of action remain incompletely understood.

Previous research has investigated the anti-tumor mechanism of ethanolic extract of Xiao-ai-fei honey ointment (XAFHO) in gastric cancer by using network pharmacology analysis [7], suggesting that XAFHO may exert therapeutic effects through multi-component and multi-target interactions. However, that study mainly focused on target–pathway prediction and did not address the cellular localization of key targets, inter-cellular communication, or the biological context of the tumor microenvironment. In addition, direct functional validation remained limited. Therefore, whether XAFHO exerts similar multi-target effects on cervical cancer and how these effects are related to tumor cells, immune cells, and patient prognosis remain unclear.

In the present study, we extended this line of investigation to cervical cancer and integrated network pharmacology with single-cell RNA sequencing, prognostic modeling, immune microenvironment analysis, molecular docking, and in vitro evaluation. By doing so, we aimed not only to identify potential targets of XAFHO but also to clarify their cellular distribution, functional relevance, and possible roles in cervical cancer progression. This is expected to provide a theoretical foundation for the development of novel therapeutic strategies for cervical cancer.

## 2. Results

### 2.1. Identification of Differentially Expressed Genes (DEGs)

**Figure 1:**
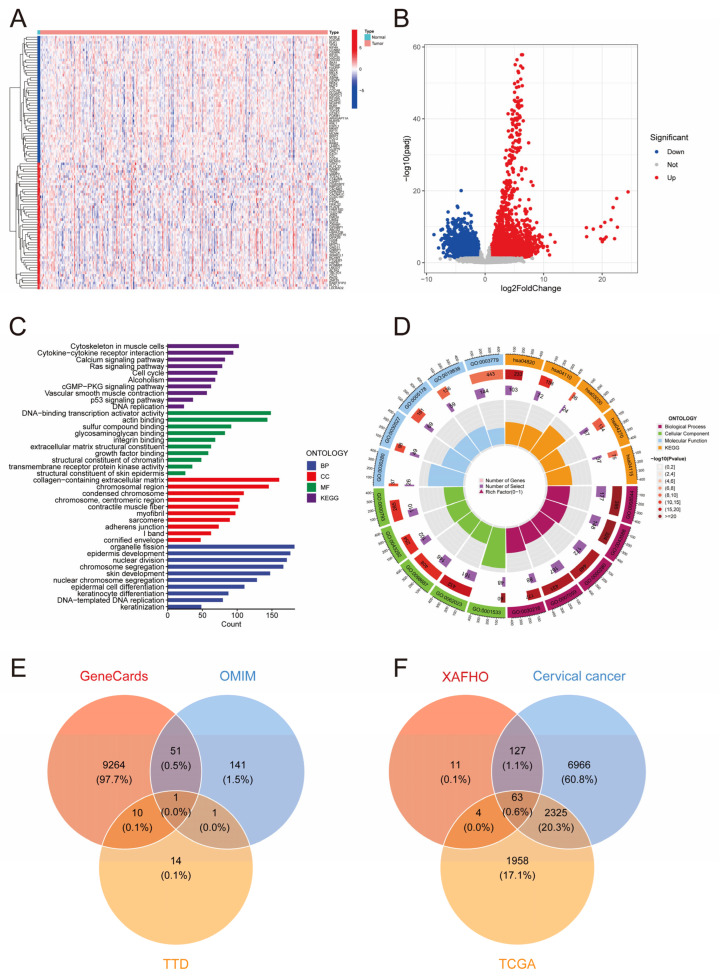
Identification and functional enrichment analysis of differentially expressed genes in cervical cancer. (**A**) Heatmap of differentially expressed genes. The top 50 up- and downregulated genes between cervical cancer and normal tissues are shown. (**B**) Volcano plot of differentially expressed genes. A total of 4350 DEGs (2672 upregulated and 1678 downregulated) are displayed. (**C**) Functional enrichment analysis of DEGs (GO and KEGG). (**D**) Circle plot of functional enrichment analysis for DEGs. This circle plot provides an overview of the enrichment of DEGs in cervical cancer across the three GO categories (biological process, BP; cellular component, CC; molecular function, MF) and KEGG pathways. (**E**) Venn diagram illustrating the intersection of cervical cancer-related targets retrieved from GeneCard, OMIM, and the TTD. A total of 9481 disease-associated targets were obtained after taking the union. (**F**) Venn diagram showing the intersection of differentially expressed genes (DEGs) from TCGA, XAFHO active ingredient targets, and cervical cancer disease targets. Sixty-three overlapping targets were identified, corresponding to 26 active components of XAFHO, suggesting that XAFHO may exert anti-cervical cancer effects by modulating these core targets.

In this study, the cervical cancer transcriptome dataset from TCGA database was acquired to elucidate the changes in gene expression profiles associated with cervical cancer onset and progression. Rigorous preprocessing steps, such as normalization and standardization, were applied to the raw transcriptome data to minimize technical variability, batch effects, and outliers, ensuring comparability of gene expression levels across samples. Subsequently, utilizing the high-quality preprocessed data, a differential expression analysis between tumor and normal tissues was performed using the DESeq2 package in R. By applying the criteria of |log2FC| ≥ 1 and padj < 0.05, 4350 DEGs were identified, with 2672 genes being upregulated and 1678 genes being downregulated significantly in cervical cancer tissues compared with normal tissues (Figure 1B). To visualize the most significantly altered genes, the top 50 upregulated and top 50 downregulated genes were selected for heatmap analysis (Figure 1A).

Subsequently, we performed enrichment analysis on the identified genes to elucidate their biological functions and potential regulatory pathways. The results of the enrichment analysis revealed significant enrichment of KEGG pathways related to the cell cycle and the p53 signaling pathway, indicating the involvement of the screened genes in the development and advancement of cervical cancer through the regulation of these pathways (Figure 1C,D). In terms of biological processes (BPs), notable enrichment was observed in functions like keratinocyte differentiation and epidermis development (Figure 1C,D), which play crucial roles in shaping the tumor microenvironment. The dysregulation of interactions among epithelial cells, immune cells, and stromal cells in the tumor microenvironment is a key factor driving tumor progression. This indicates that the identified differentially expressed genes may impact the malignant characteristics of cervical cancer by modulating inter-cellular signaling within the tumor microenvironment.

### 2.2. Acquisition of Active Components and Disease Targets of XAFHO and Intersection Analysis

**Figure 2:**
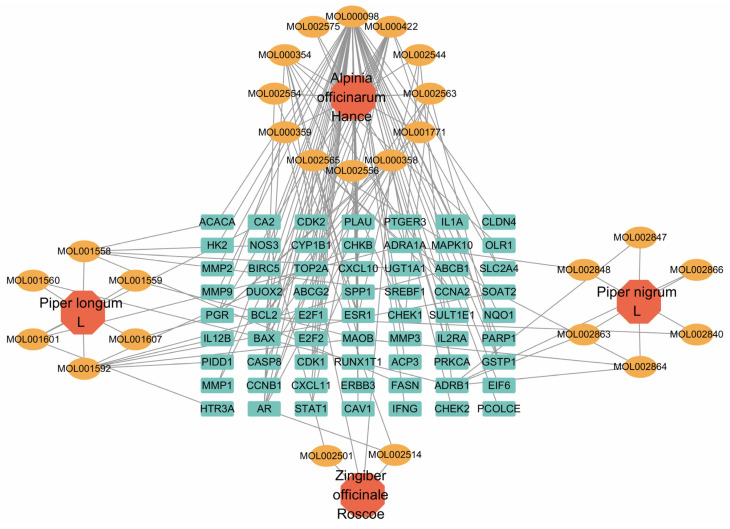
XAFHO drug–ingredient–target network. Orange nodes represent bioactive constituents of the drug, and green nodes denote target proteins. Network analysis identifies the top three compounds with the highest degree values: MOL000098 (quercetin), MOL000422 (kaempferol), and MOL000354 (isorhamnetin).

To elucidate the material basis and potential targets of XAFHO in its anti-cervical cancer effects, all chemical components of XAFHO were initially retrieved from the TCMSP database. Oral bioavailability (OB) and druglikeness (DL) are critical indicators for assessing the druggability of compounds. Therefore, this study established OB ≥ 30% and DL ≥ 0.18 as the screening criteria for active components. Subsequently, 37 effective components of XAFHO meeting these criteria were identified, corresponding to 205 component-related targets. Disease targets related to cervical cancer were obtained by simultaneously searching three target databases using “cervical cancer” as the keyword, namely, GeneCard, OMIM, and TTD. The search outcomes revealed 9325, 194, and 26 targets from GeneCard, OMIM, and the TTD, respectively. After eliminating duplicates and overlapping these targets, a total of 9481 cervical cancer-associated targets were ultimately obtained. To pinpoint potential core targets responsible for the anti-cervical cancer effects of XAFHO, an intersection analysis was conducted among the DEGs retrieved from TCGA database, the targets of active ingredients in XAFHO, and the amalgamated cervical cancer disease targets. A total of 63 intersection targets were identified, corresponding to 26 active ingredients of XAFHO, indicating that XAFHO may exert its anti-cervical cancer effects by modulating these 63 targets (Figure 1F and Table S1). Subsequently, a “drug–ingredient–target” visualization network was constructed using Cytoscape software (version 3.10.4) (Figure 2). Topological analysis of the network revealed that the three components with the highest degree values were MOL000098 (quercetin), MOL000422 (kaempferol), and MOL000354 (isorhamnetin). Notably, quercetin and kaempferol are not only network-predicted key components but also experimentally supported constituents of XAFHO, whereas isorhamnetin was identified as a candidate bioactive compound based on network pharmacology screening [21].

### 2.3. Construction and Validation of Prognostic Models

**Figure 3:**
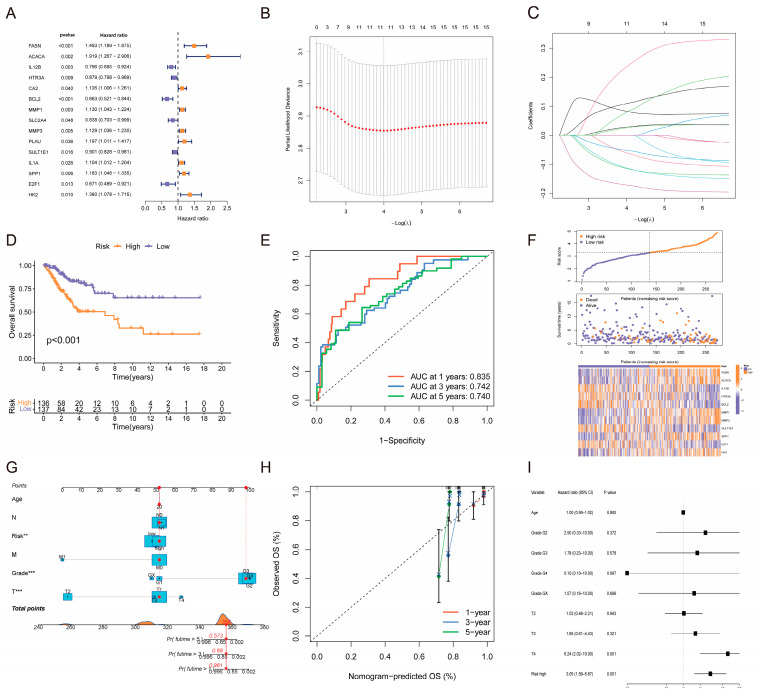
Construction, validation, and independent analysis of a cervical cancer prognostic model based on LASSO–Cox regression. (**A**) Forest plot showing the results of multivariate Cox regression analysis for the 63 intersection genes. Hazard ratios (HRs) with 95% confidence intervals (CIs) are displayed. (**B**) Selection of the optimal λ (lambda) value via 10-fold cross-validation. The dotted vertical line indicates the λ value that yields the minimum mean cross-validated error. (**C**) LASSO coefficient profiles of the 15 prognostic-related genes. Each curve represents the coefficient trajectory of a gene across different penalty parameters (λ). (**D**) Kaplan–Meier survival curves comparing OS between the high-risk and low-risk groups stratified by the prognostic model. Log-rank test *p*-value is indicated. (**E**) Time-dependent ROC curves demonstrating the predictive accuracy of the model for 1-year, 3-year, and 5-year overall survival. AUCs are displayed. (**F**) Distribution of patient risk scores (top), survival status (middle), and heatmap of the expression levels of the 11 model genes (bottom) across the high-risk and low-risk groups. (**G**) Nomogram integrating the 11-gene risk score to predict the probability of 1-year, 3-year, and 5-year OS. ** *p* < 0.01; *** *p* < 0.001. (**H**) Calibration curves comparing predicted and observed survival probabilities at 1, 3, and 5 years. The 45° line indicates perfect concordance. (**I**) Forest plot of multivariate Cox regression analysis assessing the independent prognostic value of the risk score after adjustment for clinical covariates (age, grade, and T stage). HRs and 95% CIs are shown.

To investigate the association between the mentioned intersecting genes and cervical cancer patient prognosis, we initially conducted multivariate Cox proportional hazards regression analysis on the 63 intersecting genes to identify independent prognostic factors for cervical cancer. The analysis revealed 15 genes as independent prognostic markers for cervical cancer (Figure 3A), comprising 9 high-risk genes (*FASN*, *ACACA*, *CA2*, *MMP1*, *MMP3*, *IL1A*, *SPP1*, and *HK2*) and 6 protective genes (*IL12B*, *HTR3A*, *BCL2*, *SLC2A4*, *SULT1E1*, and *E2F2*). High expression of risk-associated genes was linked to poorer patient outcomes, whereas high expression of protective genes was associated with better prognosis. To establish a robust prognostic gene signature for cervical cancer, we employed LASSO–Cox regression analysis for further variable selection and model development using the aforementioned 15 prognostic-related genes. Initially, a coefficient profile plot was generated to illustrate the evolving trend of the regression coefficient for each gene with the regularization parameter λ (Figure 3B). The results revealed a gradual transition of coefficients for selected crucial genes from zero to non-zero values with decreasing λ, underscoring the ability of λ to assess the contribution of each gene to prognostic predictions. Subsequently, the optimal λ value was determined through 10-fold cross-validation (Figure 3C), leading to the identification of the most predictive gene combination at the optimal λ value (λ.min). Ultimately, 11 genes were selected for the definitive prognostic risk model, with individual patient risk scores calculated as Risk Score = Σ (gene expression level × corresponding regression coefficient) (Table S2).

To assess the prognostic model’s predictive validity, patients were stratified into high-risk and low-risk categories using the median risk score, followed by a series of validation analyses. Survival analysis revealed significantly shorter overall survival in the high-risk group compared with the low-risk group (*p* < 0.05), indicating the model’s ability to differentiate between patients with distinct prognostic outcomes (Figure 3D). Time-dependent ROC curve analysis demonstrated that the model’s AUC for predicting 1-, 3-, and 5-year overall survival exceeded 0.7 (Figure 3E), affirming its accuracy in medium- to long-term prognostic forecasting. The risk score plot visually depicted the model’s predictive performance, highlighting that high-risk patients (depicted in red) experienced more deaths and shorter survival times (Figure 3F). External validation in an independent cohort showed poorer overall survival in the high-risk group (Figure S1A). ROC analysis was available only for 0.5, 1, and 2 years because of limited follow-up, and the AUC exceeded 0.7 at 2 years (Figure S1B), supporting the model’s value for relatively longer-term prognosis.

To operationalize this prognostic model into a personalized predictive tool for clinical use, we developed a nomogram incorporating the risk scores of 11 genes (Figure 3G) to estimate the 1-year, 3-year, and 5-year overall survival probabilities for individual patients. Evaluation through calibration curve analysis demonstrated the nomogram’s accurate calibration in predicting survival rates at these time points, aligning closely with observed patient outcomes (Figure 3H), affirming its reliability. In assessing the independence of the 11-gene risk score from conventional clinicopathological factors, a multivariate Cox proportional hazards regression model was employed, incorporating the risk score alongside factors such as age, tumor grade, and T stage. The analysis (Figure 3I) revealed that following adjustment for all variables, only T4 stage (*p* = 0.001) and high-risk score (*p* = 0.001) emerged as autonomous prognostic indicators for overall survival in cervical cancer patients, with other factors exhibiting no statistical significance (*p* > 0.05). In summary, the 11-gene prognostic risk score established in this study not only correlates significantly with cervical cancer patient prognosis but also, crucially, exhibits predictive autonomy from the traditional clinical staging system.

### 2.4. Distinct Tumor Immune Microenvironment Characteristics Between Risk Subgroups

**Figure 4:**
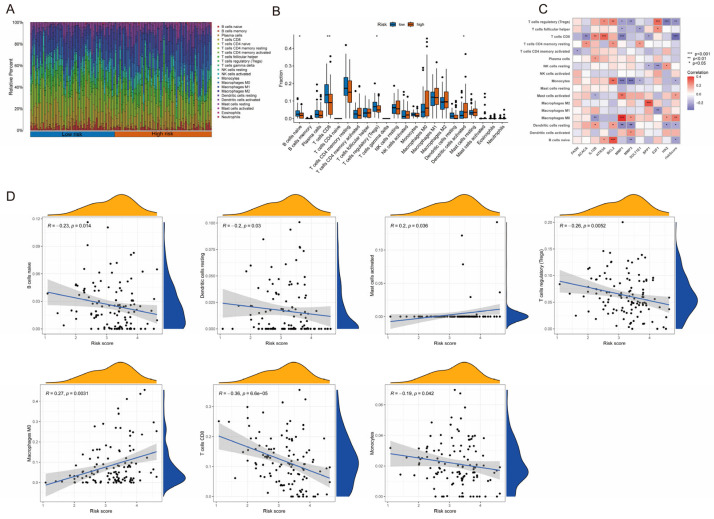
Analysis of differences in tumor immune microenvironment infiltration characteristics between different risk groups. (**A**) Stacked bar plot showing the relative proportions of 22 immune cell types in each cervical cancer sample from the high-risk and low-risk groups, as estimated by the CIBERSORT algorithm. (**B**) Violin plots comparing the infiltration levels of key immune cell subsets between the high-risk and low-risk groups. Significant differences are marked (* *p* < 0.05, ** *p* < 0.01). (**C**) Heatmap displaying the Spearman correlation coefficients between the expression levels of the 11 prognostic signature genes, the calculated risk score, and the infiltration scores of the 22 immune cell types. Red indicates positive correlation, and blue indicates negative correlation. (**D**) Scatter plots illustrating the significant negative correlations between the overall risk score and the infiltration levels of immune cells. Each dot represents a patient sample, with the trend line, correlation coefficient (r) and *p*-value being shown.

To investigate the heterogeneity of the tumor immune microenvironment (TIME) across different risk groups, we employed the CIBERSORT algorithm to conduct a deconvolution analysis utilizing tumor transcriptome data. This approach allowed us to quantitatively assess the infiltration proportions of 22 immune cell types in samples from each group, thereby elucidating the differences in immune infiltration characteristics between high- and low-risk groups. The findings indicated that cervical cancer tissues in the high-risk group displayed a pronounced immunosuppressive microenvironment phenotype (Figure 4A,B). In comparison to the low-risk group, the high-risk group exhibited significantly reduced infiltration levels of key effector immune cells, including B cells, CD8^+^ T cells, and regulatory T cells (Treg). The reduction in these cell types may compromise the body’s capacity for anti-tumor immune responses [22,23]. Conversely, the proportion of activated dendritic cells was significantly elevated in the high-risk group, and this abnormal enrichment may further exacerbate the inhibitory state of the immune microenvironment [24]. Spearman correlation analysis was performed to elucidate the relationship between the 11-gene prognostic signature and the tumor immune microenvironment. The analysis determined the correlation coefficients among gene expression levels, risk scores, and the infiltration scores of 22 immune cells. Our results revealed a significant negative correlation between the risk score and the levels of infiltrating T cells and B cells (Figure 4C,D). Particularly, the lipid metabolism genes (*FASN* and *ACACA*) integrated into the model exhibited a noteworthy correlation with immunosuppressive cells (Figure 4C). These findings suggest a close association of this prognostic signature with the development of an immunosuppressive microenvironment in cervical cancer.

### 2.5. Characterization of Somatic Mutations and Genomic Instability

**Figure 5:**
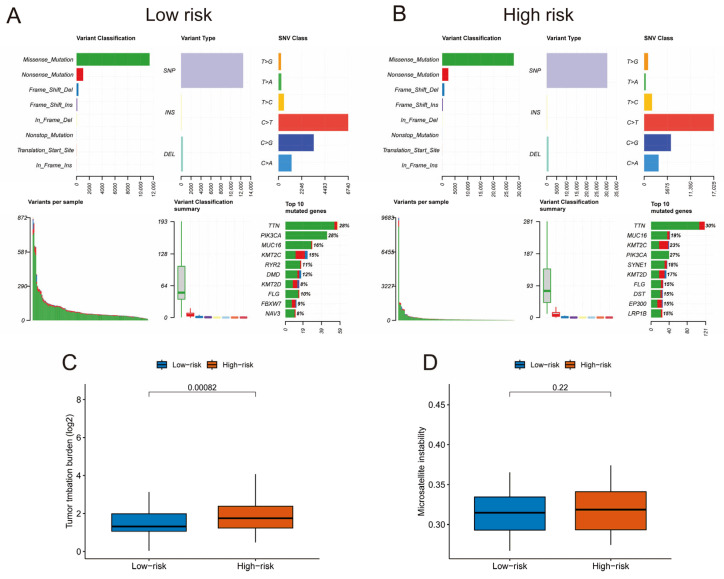
Genomic mutation landscape and microsatellite instability analysis. (**A**,**B**) Mutational landscapes of tumors in the low-risk and high-risk groups, respectively. (**C**) Box plot comparing the tumor mutational burden between the high-risk and low-risk groups. (**D**) The box plot illustrates the statistical results of microsatellite instability between the high-risk and low-risk groups. A *p*-value of less than 0.05 was considered statistically significant.

Genomic variances among different risk groups were explored through comprehensive whole-exome/targeted sequencing analysis of cohort samples, resulting in the generation of mutation landscape plots (Figure 5A,B). The plots depicted commonly mutated genes, distribution of mutation types, and TMB at the individual sample level. The analysis unveiled notably higher TMB in high-risk group patients compared with their low-risk counterparts (Figure 5C). Regarding the spectrum of mutated genes, *TTN* (mutation frequency 30%) emerged as the most frequently altered driver gene, followed by *PIK3CA*, *MUC16*, and *KMT2C*, among others, with missense mutations being the prevailing type. Furthermore, an assessment of MSI status across risk groups indicated that while the high-risk group exhibited significantly elevated TMB, its MSI status did not significantly differ from that of the low-risk group, with both primarily characterized as microsatellite-stable (Figure 5D). These results imply that the heightened TMB in high-risk tumors is not attributable to DNA mismatch repair deficiency but may stem from alternative mechanisms contributing to genomic instability.

### 2.6. Analysis of the Cervical Cancer Microenvironment at Single-Cell Resolution and Investigation into the Cellular Origin and Function of Prognostic Genes

**Figure 6:**
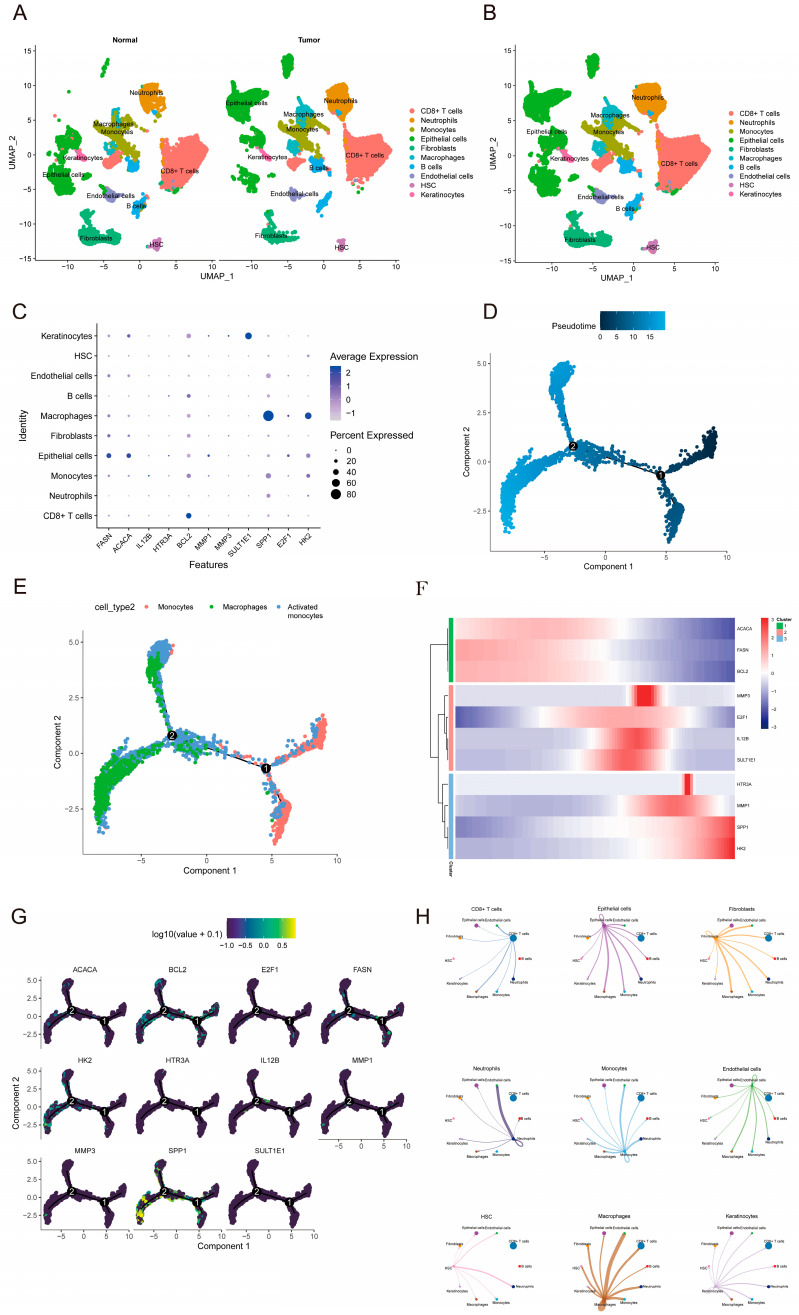
Single-cell dissection of the 11-gene signature provides insight into the mechanism of XAFHO in cervical cancer. (**A**) Uniform Manifold Approximation and Projection (UMAP) visualization of high-quality single cells obtained from the GEO dataset (GSE208653). A total of 43,493 cells were categorized into 10 major clusters based on their transcriptomic profiles. The samples were stratified into tumor and normal groups. (**B**) UMAP visualization of the combined tumor and normal group samples, in which epithelial cells also represent tumor cells. (**C**) Bubble plots depict the expression distributions of 11 prognostic models on a UMAP projection, illustrating their cell type-specific expression patterns. *FASN* and *ACACA* were predominantly highly expressed in epithelial/tumor cells, whereas *SPP1* was primarily enriched in macrophages and monocytes. (**D**,**E**) Pseudotime trajectory analysis performed on the macrophage and monocyte subpopulations. 1 and 2 denote the two trajectory branches. (**F**) Dynamic expression changes of 11 prognostic genes along the macrophage/monocyte differentiation trajectory. The expression levels of *FASN* and *ACACA* gradually decrease, while the expression of *SPP1* progressively increases during the differentiation process. (**G**) Dynamic expression changes of prognostic genes along the macrophage/monocyte differentiation trajectory. (**H**) Cell–cell communication network diagram depicting key ligand–receptor interactions among different cell subsets within the cervical cancer microenvironment.

To elucidate the tumor microenvironment at single-cell resolution and identify the cellular origins of the 11 prognostic genes, we obtained single-cell RNA sequencing data of cervical cancer from the GEO database. A total of 43,493 high-quality cells were acquired and clustered into 10 distinct subsets, which included epithelial cells/tumor cells, CD8+ T cells, B cells, macrophages, and fibroblasts (Figure 6A,B). Expression analysis revealed specific high expression of *FASN* and *ACACA* from the prognostic model in the epithelial cells/tumor cells cluster, while *SPP1* was predominantly derived from macrophages and monocytes (Figure 6C). Cell communication analysis further demonstrated that epithelial cells/tumor cells engaged in more frequent interactions with macrophages, monocytes, and CD8+ T cells via the TGFB1–TGFβR1_R2 ligand–receptor pair, potentially establishing a self-reinforcing immunosuppressive network (Figure 6H). Pseudotime analysis was conducted to investigate the functional states and differentiation trajectories of these cell subpopulations. The results revealed distinct differentiation paths for both macrophages and monocytes (Figure 6D,E). Dynamic changes in the expression of *FASN*, *ACACA*, and *SPP1* were observed during the differentiation process, with *FASN* and *ACACA* expression gradually decreasing, while *SPP1* expression progressively increased (Figure 6F). These findings suggest that these genes not only act as markers for specific cell types but also play a role in regulating the functional differentiation process of the cells.

### 2.7. Effects of XAFHO on the Malignant Biological Behavior of Cervical Cancer Cells

**Figure 7:**
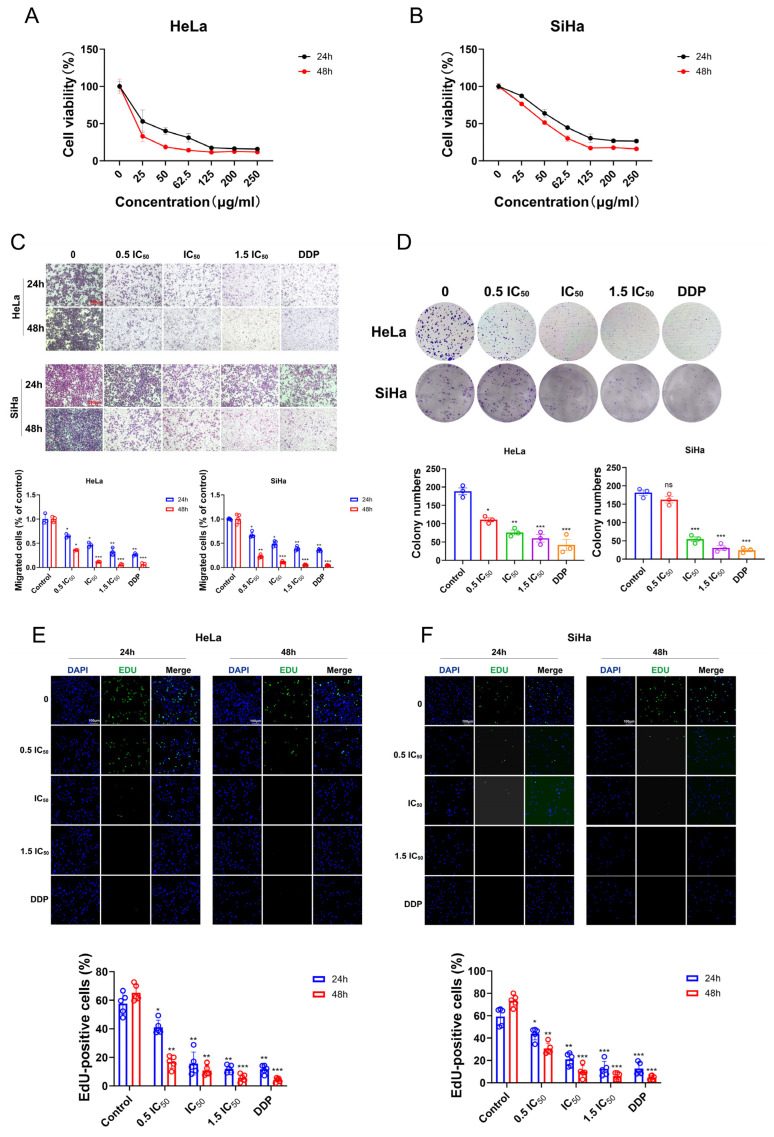
XAFHO suppresses malignant biological behaviors of cervical cancer cells. (**A**,**B**) Cytotoxicity of XAFHO against HeLa (**A**) and SiHa (**B**) cells was assessed by CCK-8 assay 24 and 48 h post-treatment, and IC50 values were calculated. The IC_50_ values for HeLa and SiHa are 30.0 ± 1.67 and 55.2 ± 2.45 μg/mL respectively. (**C**) Transwell migration assays were performed to evaluate the migratory capacity of HeLa and SiHa cells following 24 h and 48 h treatment with XAFHO at indicated concentrations (0.5 IC_50_, IC_50_, and 1.5 IC_50_). Representative images are shown (scale bar: 100 μm). Lower panels: quantitative analysis of migrated cells per field. (**D**) Colony formation assays were conducted to examine the long-term proliferative potential of HeLa and SiHa cells after XAFHO treatment for 14 days. Lower panels: quantitative analysis of colony formation rates. (**E**,**F**) EdU incorporation assays were performed to assess DNA synthesis activity in HeLa (**E**) and SiHa (**F**) cells treated with XAFHO for 24 h (EdU-positive nuclei, green; Hoechst-stained nuclei, blue). Lower panels: quantitative analysis of EdU-positive cell rates. All data are presented as means ± SDs. * *p* < 0.05, ** *p* < 0.01, and *** *p* < 0.001 vs. control group.

The anti-cervical cancer activity of XAFHO was evaluated by determining its IC50 against HeLa and SiHa cells using the CCK-8 assay. The 24 h IC_50_ values for SiHa cells were 30.0 ± 1.67 and 55.2 ± 2.45 μg/mL, respectively (Figure 7A,B). For subsequent experiments, three concentrations were selected, 0.5 IC_50_, IC_50_, and 1.5 IC_50_, with DDP serving as a positive control. Treatment with XAFHO at IC_50_ and 1.5 IC_50_ concentrations significantly inhibited the colony-forming ability of both HeLa and SiHa cells compared with the control, as shown in colony formation assays (Figure 7D). EdU incorporation assays indicated a dose-dependent decrease in the percentage of EdU-positive cells upon XAFHO treatment, suggesting effective suppression of DNA replication and cell proliferation (Figure 7E,F). Transwell invasion and migration assays demonstrated a marked reduction in the number of cells penetrating Matrigel-coated or uncoated membranes following XAFHO treatment (Figure 7C and Figure S2). In vitro, XAFHO effectively suppressed malignant cellular behaviors, including proliferation, colony formation, migration, and invasion, in cervical cancer cells.

### 2.8. Molecular Docking Analysis

**Figure 8:**
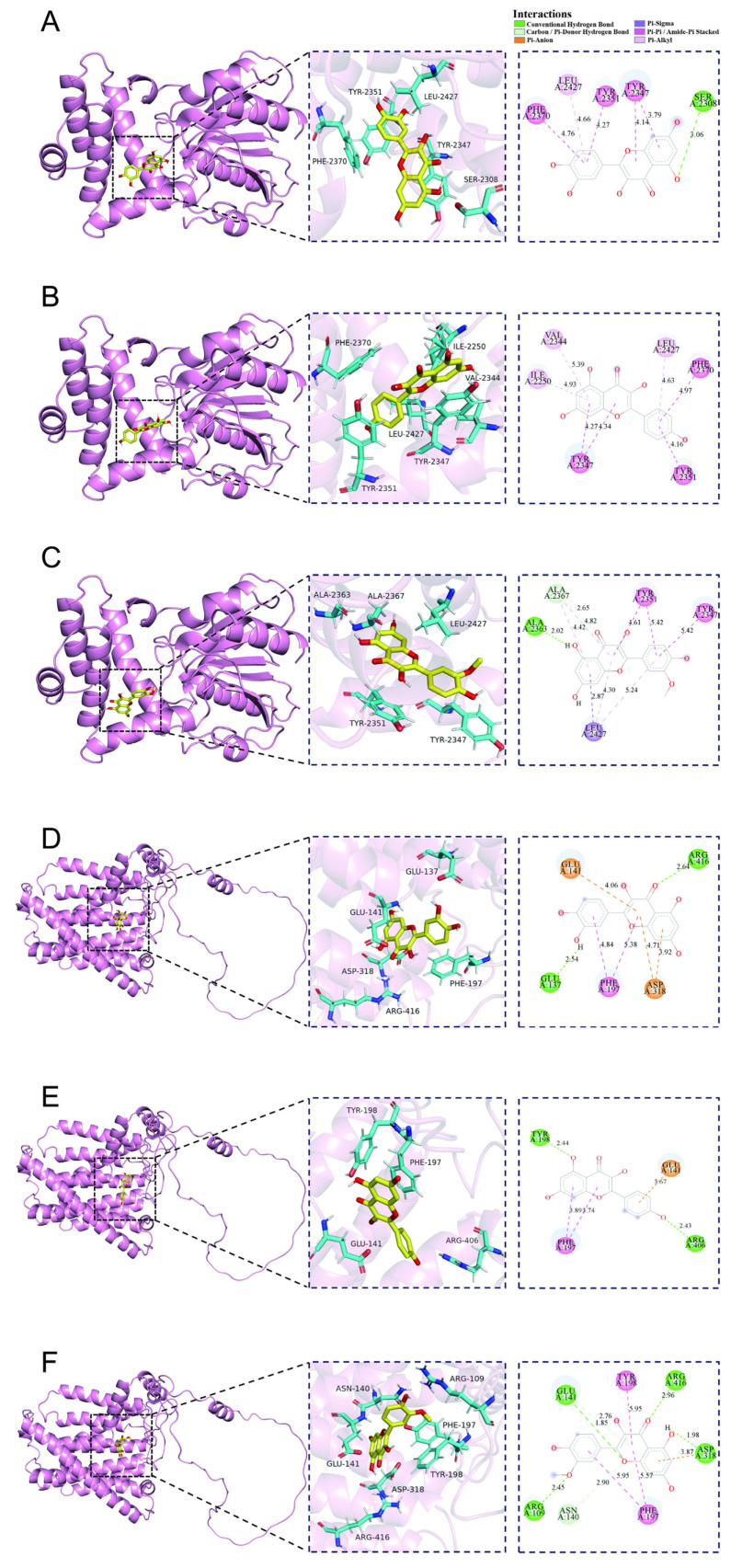
Molecular docking models. (**A**) Quercetin with *FASN*. (**B**) Kaempferol with *FASN*. (**C**) Isorhamnetin with *FASN*. (**D**) Quercetin with *SPP1*. (**E**) Kaempferol with *SPP1*. (**F**) Isorhamnetin with *SPP1*.

To further explore the potential interactions between XAFHO and key targets in cervical cancer, we selected the three compounds (Quercetin, Kaempferol, and Isorhamnetin) with the highest median XAFHO values for molecular docking analysis. *FASN* and *SPP1* were selected as docking targets because both were prognostic risk genes with distinct biological relevance in cervical cancer. Single-cell analysis showed that *FASN* was enriched in tumor cells, whereas *SPP1* was enriched in monocytes/macrophages. Additionally, *SPP1* has been reported to be highly expressed in cervical cancer and related to immunotherapy response [25]. These three compounds exhibited strong binding affinities with both *FASN* and *SPP1*, which are prognostic genes. All binding energies were below -6.0 Kcal/mol, suggesting potential binding affinity between representative XAFHO components and these target proteins (Figure 8A–F).

### 2.9. The Impact of the Knockdown of FASN and SPP1 on the Biological Behavior of Cervical Cancer Cells

**Figure 9:**
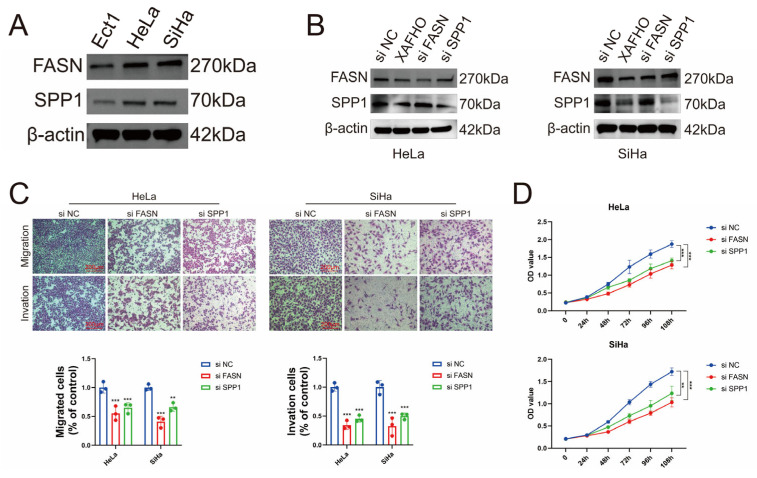
*FASN* and *SPP1* knockdown suppresses cervical cancer cell malignancy. (**A**) Expression levels of *FASN* and *SPP1* in HeLa and SiHa cervical cancer cell lines compared with normal cervical epithelial cells line Ect1/E617, as determined by Western blot analysis. (**B**) Western blot analysis showing that treatment with XAFHO at the IC_50_ concentration for 24 h decreased *FASN* and *SPP1* protein expression in HeLa and SiHa cells, respectively, while siRNA-mediated knockdown effectively reduced their expression. (**C**) Effects of *FASN* or *SPP1* knockdown on the migration and invasion capacities of HeLa and SiHa cells, as assessed by transwell assays. (**D**) Effects of *FASN* or *SPP1* knockdown on the proliferation of HeLa and SiHa cells, as evaluated by CCK-8 assays. All data are presented as means ± SDs. ** *p* <0.01 and *** *p* < 0.001 vs. si NC.

To evaluate *FASN* and *SPP1* as key targets underlying the anti-cervical cancer effects of XAFHO, we initially assessed their expression levels in cervical cancer cells. Relative to normal cervical epithelial cells, both *FASN* and *SPP1* exhibited significant upregulation in the HeLa and SiHa cervical cancer cell lines (Figure 9A). We subsequently treated cervical cancer cells with XAFHO to examine its effects on the expression of these targets. RT-qPCR analysis indicated that XAFHO treatment did not significantly change the mRNA levels of *FASN* or *SPP1* in HeLa or SiHa cells (Figure S3). In contrast, Western blot analysis revealed a substantial reduction in their protein expression (Figure 9B), suggesting that XAFHO may regulate *FASN* and *SPP1* at the post-translational level rather than at the transcriptional level. To further elucidate the functional roles of *FASN* and *SPP1* in cervical cancer cells, we conducted siRNA-mediated knockdown of these genes in HeLa and SiHa cell lines (Figure 9B). Functional assays demonstrated that the knockdown of either *FASN* or *SPP1* significantly inhibited cell proliferation (Figure 9D) and diminished cell migration and invasion capacities (Figure 9C). These findings suggest that XAFHO may exert its anti-cancer effects, at least in part, through the downregulation of *FASN* and *SPP1* at the protein level.

## 3. Discussion

This study combined network pharmacology, single-cell RNA sequencing, and molecular docking to investigate the anti-cervical cancer mechanism of XAFHO, a traditional Uyghur medicinal formulation. Our analysis revealed 37 active compounds and 63 essential anti-cervical cancer targets of XAFHO. An independent 11-gene prognostic model was established, with an AUC > 0.7 for predicting 1–5-year survival. The high-risk group exhibited a more immunosuppressive tumor microenvironment and higher TMB. Single-cell analysis elucidated the cellular localization of core genes and inter-cellular communication networks within the tumor microenvironment. In vitro experiments also verified the effectiveness of the drug. Molecular docking showed the stable binding (binding energy < −6.0 Kcal/mol) between XAFHO’s active components and key targets. Functional validation showed that *FASN* and *SPP1* were significantly upregulated in cervical cancer cells, and silencing either target markedly suppressed malignant cellular behaviors. In conclusion, XAFHO exerts its anti-cervical cancer effects through the regulation of multiple components, targets, and cells.

Previous research explored the anti-gastric cancer mechanism of XAFHO by using network pharmacology [7]. However, such analyses mainly predict target–pathway relationships and do not adequately address cellular localization, inter-cellular communication, or dynamic regulation within the tumor microenvironment, thereby limiting direct biological validation [26]. This study integrates network pharmacology with single-cell sequencing to not only identify core targets but also validate through single-cell analysis that *FASN* and *ACACA* exhibit high expression in epithelial/tumor cells, whereas SPP1 primarily originates from macrophages and monocytes. Additionally, epithelial/tumor cells interact extensively with macrophages, monocytes, and CD8+ T cells through the TGFB1–TGFβR1_R2 ligand–receptor pair, establishing an immunosuppressive network. Furthermore, dynamic alterations in gene expression during cell differentiation were observed. Compared with network pharmacology alone, this integrated approach provides a more comprehensive view of the multi-cellular anti-tumor mechanism of XAFHO by linking tumor cells, myeloid cells, and inter-cellular signaling networks.

Building on our integrated analysis, the 11-gene prognostic model and its associated immune and genomic features provide further insight into the molecular basis of XAFHO’s anti-cervical cancer effects. The immunosuppressive tumor immune microenvironment (TIME) plays a critical role in the progression of cervical cancer by impairing the infiltration of effector immune cells and promoting inhibitory cell subsets [27,28,29]. This “cold” TIME profile is associated with decreased B-cell and CD8+ T-cell infiltration, alongside an increase in Tregs, collectively impairing anti-tumor cytotoxicity and contributing to the immune evasion and poor prognosis observed in cervical cancer [30,31]. In conjunction with higher TMB in the high-risk group, this immunosuppressive environment may reduce the effectiveness of monotherapy with immune checkpoint inhibitors, although further validation is needed. Thus, this prognostic model serves as a useful risk stratification tool, identifying high-risk patients who may benefit from combined therapeutic strategies, though the effectiveness of specific combinations requires further investigation. Furthermore, considering that the key active constituents of XAFHO (Quercetin, Kaempferol, and Isorhamnetin) target *FASN* and *SPP1*, which are markedly upregulated in this high-risk context, XAFHO could be considered a promising candidate for combination therapy, potentially enhancing the effects of immunotherapies (e.g., PD-1/PD-L1 inhibitors) by targeting tumor metabolic pathways (via *FASN*/*ACACA*) and reversing immunosuppression (via *SPP1*), though preclinical validation is required to confirm these effects. Subsequent preclinical investigations are essential to validating this combined treatment strategy and to assess the utility of this 11-gene signature as a predictive biomarker for treatment response.

Our genomic analysis revealed the complex molecular landscape associated with high-risk tumors, including factors related to the immune microenvironment. TMB, an indicator of genomic instability, has been suggested to interact with the immune microenvironment in cervical cancer [32,33]. High TMB is associated with increased production of neoantigens in cervical cancer, which could theoretically enhance immune recognition of tumor cells [33,34,35]. However, within an immunosuppressive TIME, these neoantigens may not effectively trigger anti-tumor immune responses, potentially leading to immune evasion in cervical cancer [35]. Consistent with previous studies, our genomic analysis showed that high-risk group cervical cancer patients exhibited significantly elevated TMB compared with those in the low-risk group, suggesting that TMB may influence the prognosis and treatment response in cervical cancer patients. Interestingly, there was no notable distinction in MSI status between the two groups, implying that the high TMB was not attributable to DNA mismatch repair deficiency.

The core prognostic genes are functionally involved in pathways crucial to tumor progression. *FASN* and *ACACA* represent pivotal enzymes in the de novo fatty acid synthesis pathway [36]. Previous studies have identified *FASN* as a critical driver of metabolic reprogramming and treatment resistance in cervical cancer, and pharmacological inhibition of *FASN* has been reported to restore cisplatin sensitivity in resistant models [37,38,39,40]. Likewise, *SPP1* has been implicated in cervical cancer invasiveness, immune evasion, and responsiveness to PD-1 blockade, suggesting that it plays an important role in shaping the tumor immune microenvironment [25,41]. These findings indicate that both *FASN* and *SPP1* are not merely correlated with malignant progression but may function as actionable targets in cervical cancer. Consistent with these previous observations, our study further supports the pathological relevance of *FASN* and *SPP1* in cervical cancer. We found that both genes were significantly upregulated in HeLa and SiHa cells compared with normal cervical epithelial cells, which is in agreement with the reported oncogenic roles of these targets. Functionally, siRNA-mediated knockdown of either *FASN* or *SPP1* significantly suppressed cervical cancer cell proliferation, migration, and invasion, further indicating that both molecules contribute to the malignant phenotype. Molecular docking reveals the stable binding of quercetin, kaempferol, and isorhamnetin to *FASN* and *SPP1*, with binding energies below −6.0 kcal/mol, supporting the hypothesis that XAFHO’s active components modulate lipid metabolism pathways and the immune microenvironment by targeting *FASN* and *SPP1* to exert anti-cervical cancer effects. Importantly, quercetin and kaempferol are supported by both experimental evidence and network analysis, enhancing consistency between chemical characterization and prediction, whereas others (e.g., isorhamnetin) remain putative candidates requiring further validation.

This study, through the combination of computational biology and single-cell omics techniques, provides a systematic theoretical framework for the multi-target and multi-cell synergistic mechanism of XAFHO against cervical cancer. However, some limitations still need to be addressed in future work. First, animal models are needed for further in vivo verification. Second, although our results support the potential involvement of *FASN* and *SPP1* at the protein and functional levels, their precise signaling pathways and regulatory mechanisms have not yet been fully characterized and will require further pathway-level validation in future studies. In addition, due to the complex composition of traditional compound preparations and variable metabolic processes, they cannot be fully simulated through computational models. Therefore, the molecular docking results should be considered preliminary and require experimental validation. Future research should combine medicinal chemistry and pharmacokinetic methods to isolate and identify the active components in vivo and clarify their pharmacodynamic basis. Although this study mainly focuses on static network pharmacology to analyze drug–target interactions, the dynamic regulation of the disease network—including XAFHO’s impact on edge perturbation and module regulation—is a key area for future research. This dynamic analysis is crucial to fully understanding XAFHO’s therapeutic potential.

## 4. Materials and Methods

### 4.1. Data Acquisition

Gene expression data, clinical data, and somatic mutation data for cervical cancer were downloaded from The Cancer Genome Atlas (TCGA) database (https://portal.gdc.cancer.gov/, accessed on 15 May 2025). Single-cell RNA sequencing (scRNA-seq) dataset GSE208653 was retrieved from the Gene Expression Omnibus (GEO) database (https://www.ncbi.nlm.nih.gov/geo/, accessed on 15 May 2025), and five samples (GSM6360682, GSM6360683, GSM6360686, GSM6360687, and GSM6360688) were selected for subsequent single-cell analysis.

### 4.2. Acquisition of Drug Components and Targets

The active constituents of the compound drug XAFHO were sourced from the Traditional Chinese Medicine Systems Pharmacology Database and Analysis Platform (TCMSP). Screening criteria included oral bioavailability (OB) ≥ 30% and druglikeness (DL) ≥ 0.18 to identify potential active components of XAFHO. Subsequently, the potential therapeutic targets corresponding to the screened active components were retrieved from the TCMSP database. Disease targets associated with cervical cancer were collected from the GeneCard database, Online Mendelian Inheritance in Man (OMIM) database, and the Therapeutic Target Database (TTD) using the search term “cervical cancer.” A relevance score > 5 from the GeneCard database was used as the screening threshold. Ultimately, targets from the three databases were amalgamated to constitute the cervical cancer disease target set.

### 4.3. Differential Gene Identification and Enrichment Analysis

R software (version 4.5.1) was employed to preprocess and analyze the raw RNA-seq count data, aiming to remove batch effects and random noise for ensuring data reliability. Differential expression analysis was conducted using the DEseq2 package, with the criteria set at |log2 fold change (log2FC)| > 1 and adjusted *p*-value (Padj) < 0.05 to identify differentially expressed genes (DEGs) between cervical cancer tissues and normal tissues. Subsequently, gene functional enrichment analysis was carried out utilizing the clusterProfiler package, encompassing Gene Ontology (GO) functional enrichment analysis (encompassing biological process, cellular component, and molecular function) and Kyoto Encyclopedia of Genes and Genomes (KEGG) pathway enrichment analysis, with a significance threshold of Padj < 0.05. The visualization of the analysis results involved the use of the ggplot2 package and enrichplot package to generate volcano plots, bar plots, bubble plots, and enrichment networks, facilitating an intuitive display of the characteristics of DEGs and enrichment outcomes.

### 4.4. Identification of Prognostic Genes

The active components in XAFHO, disease targets related to cervical cancer, and differentially expressed genes from TCGA database were analyzed to identify a key gene set for drug intervention in cervical cancer. Prognostic correlation analysis was conducted on this gene set using the survival and survminer packages in R software. Patients were categorized into high-expression and low-expression groups based on median gene expression levels, with overall survival (OS) as the primary outcome. The survival disparity between the groups was assessed using the log-rank test, identifying genes significantly linked to patient prognosis (*p* < 0.05 denotes significance). Subsequently, Cox proportional hazards regression analysis was employed to confirm and pinpoint independent prognostic genes. Initially, univariate Cox regression analysis was utilized to screen genes associated with patient OS (*p* < 0.1 for inclusion). These genes were then subjected to multivariate Cox regression to adjust for variables like age and clinical stage, ultimately revealing the prognostic genes.

### 4.5. Construction of the Gene-Associated Prognostic Model

To refine the gene set, eliminate false-positive results, and mitigate overfitting in the subsequent model, Lasso regression analysis was conducted as a secondary screening of the prognostic-related genes identified through Cox regression analysis. The glmnet package in R was utilized for this analysis, employing 10-fold cross-validation to ascertain the optimal penalty parameter λ (lambda). A core gene set, strongly associated with cervical cancer prognosis, was selected based on the principle of minimum deviation. Core genes identified through Lasso regression were incorporated into a multivariate Cox model, adjusting for confounding factors such as age and stage, to pinpoint independently predictive genes (*p* < 0.05). mRNA expression data for these genes in cervical cancer and adjacent normal tissues were extracted from TCGA database to validate expression differences and confirm clinical relevance. A cervical cancer risk scoring model was developed using independent prognostic genes from Cox regression analysis and their coefficients, calculating each patient’s risk score through a linear combination of gene expression levels and β values. The calculation formula is as follows:

Prognostic model/Risk Score = β_1_ × gene_1_ + β_2_ × gene_2_ +… + β_n_ × gene_n_

### 4.6. Validation of the Prognostic Model

TCGA cervical cancer cohort was stratified into high-risk and low-risk groups according to median risk scores. Kaplan–Meier survival analysis was performed to compare overall survival (OS) using log-rank tests. The pROC package was utilized to generate ROC curves and calculate AUC values for 1-year, 3-year, and 5-year survival, with an AUC greater than 0.7 indicating good predictive accuracy. Furthermore, pheatmap was employed to visualize risk scores and differences in gene expression. A prognostic nomogram, developed using the rms package, predicted survival rates based on risk scores and clinical features. Lastly, calibration curves were used to evaluate model consistency against actual survival durations.

### 4.7. Immune Infiltration and Mutation Analysis

CIBERSORT (https://github.com/, accessed on 1 June 2025) analyzed the ratios of immune cell infiltration, establishing correlations between signature gene expression and immune cell infiltration in cervical cancer. The tumor mutational burden (TMB) was determined using mutation data from The Cancer Genome Atlas (TCGA) to compare high-risk and low-risk groups while also examining the distribution of microsatellite instability (MSI) status to evaluate disparities in genomic instability.

### 4.8. Single-Cell RNA Sequencing Analysis

The GSE208653 dataset served as the basis for single-cell analysis in this study. The Seurat package facilitated routine single-cell data processing, encompassing several steps: data preprocessing and filtering, during which low-quality cells exhibiting excessively high mitochondrial gene ratios, abnormal ribosomal gene ratios, and irregular gene/UMI counts per cell were excluded; subsequent normalization and standardization of the data aimed to mitigate technical variations across samples; Principal Component Analysis (PCA) was employed for initial dimensionality reduction, with key principal components being selected for further investigation. Additionally, the UMAP algorithm was utilized for visual dimensionality reduction of high-dimensional data, followed by a graph-based clustering algorithm to categorize cells. Cell–cell communication analysis was conducted using the CellChat package. By constructing inter-cellular signaling pathway networks, this study quantified communication intensity and identified key signaling molecules among different cell subsets, thereby elucidating interaction patterns within the cervical cancer microenvironment. Pseudotime analysis was carried out with the Monocle package, which traced differentiation paths and dynamic changes of various cell subsets by constructing cell development trajectories, thereby clarifying the state transition rules of cells throughout disease progression. Ultimately, distinct cell subsets were identified, and cell type annotation was completed using singleR.

### 4.9. Preparation of XAFHO

*Piper longum L* (Batch No. 231140132), *Alpinia officinarum Hance* (Batch No. Q30025805), *Piper nigrum L* (Batch No. M30067608), *Zingiber officinale Roscoe* (Batch No. 240401) and *Zaocys dhumnades* (Batch No. 20250101) were kindly provided by Xinjiang Uyghur Autonomous Region Uyghur Medical Hospital (Urumqi, China). The herbs were identified by Dilixiati Siyiti, a professor of traditional Chinese medicine at Xinjiang Uyghur Autonomous Region Uyghur Medical Hospital. The composition of the formulation is shown in Table S3. Regarding the preparation of the medicinal product, specifically, the medicinal herbs were first ground into powder and then mixed with excipients (honey) to form the ointment base. We took 5 kg of the prepared ointment, which was soaked in 8 times the volume of 95% ethanol for 12 h. The mixture was heated in a water bath to 78 °C and reflux-extracted three times (3 h, 3 h, and 2 h). The mixture was then filtered while still hot through a gauze, and the filtration was combined and concentrated under reduced pressure. The concentrate was subsequently freeze-dried to remove any remaining solvent. The final extract obtained was the ethanol extract of Xiao-ai-fei honey ointment, weighing 2.25 kg (yield 45%). The known active constituents with therapeutic activity are piperine and galangin, and the genuine extract content ranges from 76% to 87%. Quercetin and kaempferol were also identified as confirmed constituents of XAFHO, and components of XAFHO were identified by GC-MS and provided by Uyghur Medical Hospital [21]. The extract was then dissolved in DMSO at a concentration of 4 mg/mL, followed by centrifugation at 12,000 rpm for 15 min to collect the supernatant. Subsequently, the supernatant was filtered using a 0.22 μm filter for future use. For application, the ointment should be diluted to the required concentration with a complete medium to make it ready for use. The material used in the in vitro experiments was the ethanolic extract of XAFHO; for simplicity, it is hereafter referred to as “XAFHO” unless otherwise specified.

### 4.10. Cell Proliferation Assays

Cell proliferation rates were assessed quantitatively using the CCK-8 assay kit by ZEN (Chengdu, China). For IC_50_ determination, cells were seeded at a density of 5 × 10^3^ cells/well in 96-well plates and then treated with various concentrations of XAFHO (0–250 μg/mL) or cisplatin. For routine proliferation assays, cells were seeded at a density of 2 × 10^3^ cells/well in 96-well plates prior to treatment. The IC_50_ value was calculated from the optical density readings at 450 nm. In the colony formation assay, cells were plated in 6-well dishes at a density of 1 × 10^3^ cells/well and incubated for 14 days. Colonies were fixed with 4% paraformaldehyde, stained with 0.1% crystal violet, and manually counted using a microscope.

### 4.11. Transwell Migration and Invasion Assays

HeLa and SiHa cells were seeded at a density of 3 × 10^5^ cells per well in the upper chamber of the transwell migration assay, with drugs introduced into the lower chamber. Subsequently, after 24 and 48 h, the migrated cells were fixed, stained with crystal violet, and quantified. In the matrigel invasion assay, the upper chambers were pre-coated with Matrigel (Corning, Corning, NY, USA) using procedures analogous to those of the migration assay.

### 4.12. EdU Assays

HeLa and SiHa cells were seeded at a density of 1 × 10^5^ cells per well in 24-well plates, cultured overnight, and then exposed to specified concentrations of test compounds for 24 or 48 h. Following treatment, the cells were subjected to a series of steps: incubation with 10 μM EdU (Elabscience, Wuhan, China) for 2 h at 37 °C, fixation with 4% paraformaldehyde for 15 min, permeabilization with 0.5% Triton X-100 for 20 min, and addition of the Click-iT reaction cocktail for 30 min in the dark. Subsequently, the cell nuclei were stained with Hoechst 33342 for 5 min. Proliferating cells, identified by EdU incorporation, were observed and captured using a fluorescence microscope.

### 4.13. Western Blot Assays

Total protein was extracted using RIPA buffer (Bioss, Beijing, China) supplemented with protease inhibitors. Protein concentrations were determined using the BCA assay kit (Biosharp, Hefei, China). Equal amounts of protein were separated by SDS-PAGE, transferred to PVDF membranes, and blocked with 6% non-fat milk. Membranes were incubated with primary antibodies overnight at 4 °C, followed by the corresponding secondary antibody (Abways, Shanghai, China, AB0103). Protein bands were visualized using the ChemiScope 6000 Touch imaging system (Clinx Science Instruments, Shanghai, China). This work utilized the following antibodies: b-actin (cat#AB0035, Abways), *SPP1* (cat#0806-6, HUABIO, Woburn, MA, USA) and *FASN* (cat#66591-1-lg, Proteintech, Rosemont, IL, USA).

### 4.14. RT-qPCR

Total RNA was extracted from cervical cancer cells using an RNA Extraction Kit (Accurate Biotechnology, Guangzhou, China, AG21023). Reverse transcription of 1 μg of total RNA was performed with the SynScript III RT SupreMix for qPCR with gDNA Remover (Tsingke, Beijing, China, TSK314M). mRNA levels were quantified using 2 × TSINGKE Master qPCR Mix (Tsingke, TSE203) on a qTOWER3 G qPCR cycler (Analytik, Jena, Germany). The primer pairs of *FASN* were forward: 5′-TGAGAGATGGCTTGCTGGA-3′; reverse: 5′-CCGCTGTACTTGGGCTTG-3′. Those of *SPP1* were forward: 5′-CACCTGTGCCATACCAGTTA-3′; reverse: 5′-TGTGTGCCTTTTTGTCCAAGC-3′. Those of *Actin* were forward: 5′-GCGAGAAGATGACCCAGATC-3′; reverse: 5′-CCAGTGGTACGGCCAGAGG-3′.

### 4.15. Cell Culture and Transfection

Cervical cancer cell lines including HeLa and SiHa and normal cervical epithelial cells (Ect1/E6E7) were acquired from West China Second University Hospital, Sichuan University (Chengdu, China). The cells were cultured in DMEM supplemented with 10% fetal bovine serum and 1% penicillin–streptomycin. Standard incubation conditions of 37 °C, 5% CO2, and humidity were consistently maintained.

For transfection experiments, we used two small interfering RNAs (siRNAs) targeting *FASN* and *SPP1* obtained from GenePharma (Shanghai, China, si-*SPP1*: 5′-CCAUGAAGAUAUGCUGGUUTT-3′; si-*FASN*:5′-GGUAGUGAGUGGGAAGGUGUATT-3′; si-NC:5′-UUCUCCGAACGUGUCACGUTT-3′). Western blot analysis confirmed knockdown efficiency 48 h after transfection. Concurrent phenotypic experiments were carried out following the same protocol. 

### 4.16. Molecular Docking

Molecular docking was performed using AutoDock Vina 1.5.7 to evaluate the binding interactions between XAFHO components and target proteins. The crystal structure of *FASN* (PDB ID: 3TJM) and the protein structure of *SPP1* (UniProt ID: Q9BX95) were obtained and preprocessed using PyMOL 3.0.3. Ligands were energy-minimized using ChemDraw 20.0, converted into PDBQT format, and prepared for docking. The docking search space (grid box) was defined as follows: for *FASN*, size_x = 61.2, size_y = 54.7, and size_z = 68.8; for *SPP1*, size_x = 70.1, size_y = 45.3, and size_z = 71.7. Docking was performed with 20 independent runs, and the conformation with the lowest binding energy was selected for further analysis. The docking results were visualized using PyMOL 3.1 and analyzed with Discovery Studio 2021. All other parameters were set to default values.

### 4.17. Statistical Analysis

Statistical analyses were performed using R and GraphPad Prism 10.1.2. Categorical variables were assessed utilizing the chi-square test or Fisher’s exact test, selected based on expected cell frequencies. Survival outcomes were evaluated via Kaplan–Meier estimation, with group comparisons conducted using the log-rank test, and multivariate Cox proportional hazards regression was carried out. For all in vitro experiments involving group comparisons, the Kruskal–Wallis test was used regardless of whether two or more groups were compared. All experiments were conducted with at least three independent biological replicates, and data are expressed as means ± standard deviations. Statistical significance was set to *p* < 0.05, with significance being denoted by asterisks: n.s. (non-significant), * *p* ≤ 0.05, ** *p* ≤ 0.01, *** *p* ≤ 0.001, and **** *p* ≤ 0.0001. *p*-Values under 0.05 were deemed statistically significant.

## 5. Conclusions

In conclusion, this study integrated network pharmacology, single-cell analysis, and in vitro experiments to explore the potential anti-cervical cancer mechanism of XAFHO. By combining transcriptomic and network analyses, we identified 11 key genes and established a prognostic model associated with overall survival, mutation patterns, and immune microenvironment characteristics in cervical cancer. Single-cell analysis further suggested distinct cellular distributions of key targets, with *FASN* being mainly enriched in tumor cells and *SPP1* being predominantly expressed in macrophages/monocytes. In vitro experiments supported the inhibitory effects of XAFHO on the malignant behaviors of cervical cancer cells, while molecular docking provided preliminary evidence that quercetin, kaempferol, and isorhamnetin may interact with *FASN* and *SPP1*. Collectively, these findings suggest that XAFHO may exert anti-cervical cancer effects through coordinated regulation of multiple components, targets, and cell types and provide a basis for further mechanistic and translational studies.

## Data Availability

The original contributions presented in this study are included in the article/Supplementary Material. Further inquiries can be directed to the corresponding authors.

## Supplementary Materials

The following supporting information can be downloaded at: https://www.mdpi.com/article/10.3390/ph19050686/s1, Figure S1: Evaluation of the Prognostic Model in the Validation Cohort. Figure S2: XAFHO Suppresses Invasion of Cervical Cancer Cells. Figure S3: Effect of XAFHO on the mRNA expression levels of *FASN* and *SPP1* in cervical cancer cells. Table S1: Information on bioactive constituents identified in CXHO. Table S2: Multivariable Cox regression analysis of the 11 prognostic genes in cervical cancer. Table S3: The composition of XAFHO.

## Abbreviations

The following abbreviations are used in this manuscript:

XAFHO	Xiao-ai-fei Honey Ointment
HPV	Human Papillomavirus
TCGA	The Cancer Genome Atlas
DEG	Differentially Expressed Gene
LASSO	Least Absolute Shrinkage and Selection Operator
TMB	Tumor Mutational Burden
TIME	Tumor Immune Microenvironment
MSI	Microsatellite Instability
FASN	Fatty Acid Synthase
SPP1	Secreted Phosphoprotein 1
TGFB1	Transforming Growth Factor Beta 1
TGFβR1/R2	Transforming Growth Factor Beta Receptor 1/2
GO	Gene Ontology
KEGG	Kyoto Encyclopedia of Genes and Genomes
BP	Biological Process
CC	Cellular Component
MF	Molecular Function
TCMSP	Traditional Chinese Medicine Systems Pharmacology Database
OB	Oral Bioavailability
DL	Druglikeness
OMIM	Online Mendelian Inheritance in Man
TTD	Therapeutic Target Database
ACACA	Acetyl-CoA Carboxylase Alpha
CA2	Carbonic Anhydrase 2
MMP1	Matrix Metallopeptidase 1
MMP3	Matrix Metallopeptidase 3
IL1A	Interleukin 1 Alpha
HK2	Hexokinase 2
IL12B	Interleukin 12B
HTR3A	5-Hydroxytryptamine Receptor 3A
BCL2	B-Cell Lymphoma 2
SLC2A4	Solute Carrier Family 2 Member 4
SULT1E1	Sulfotransferase Family 1E Member 1
E2F2	E2F Transcription Factor 2
HR	Hazard Ratio
CI	Confidence Interval
OS	Overall Survival
ROC	Receiver Operating Characteristic
AUC	Area Under the Curve
Treg	Regulatory T Cell
TTN	Titin
PIK3CA	Phosphatidylinositol-4,5-Bisphosphate 3-Kinase Catalytic Subunit Alpha
MUC16	Mucin 16
KMT2C	Lysine Methyltransferase 2C
scRNA-seq	Single-Cell RNA Sequencing
GEO	Gene Expression Omnibus
UMAP	Uniform Manifold Approximation and Projection
TGFβR1_R2	Transforming Growth Factor Beta Receptor 1 and 2
DDP	Cisplatin
IC50	Half-Maximal Inhibitory Concentration
EdU	5-Ethynyl-2‘-Deoxyuridine
PDB	Protein Data Bank
PCA	Principal Component Analysis
DMSO	Dimethyl Sulfoxide
CCK-8	Cell Counting Kit-8
DMEM	Dulbecco’s Modified Eagle Medium
FBS	Fetal Bovine Serum
PD-1	Programmed Cell Death Protein 1
PD-L1	Programmed Death-Ligand 1
