# Supplementary material for: Mechanism of Xiao-ai-fei Honey Ointment, a Traditional Uyghur Multi-Ingredient Medicinal Preparation, Against Cervical Cancer Based on Network Pharmacology and In Vitro Evaluation of Anti-Cancer Activity
Source: Pharmaceuticals (Basel). 2026 Apr 27;19(5):686. doi: 10.3390/ph19050686 (PMC13209383; doi:10.3390/ph19050686)
Supplement: Supplementary file 1 [file pharmaceuticals-19-00686-s001.zip › pharmaceuticals-4181136-supplementary.pdf]

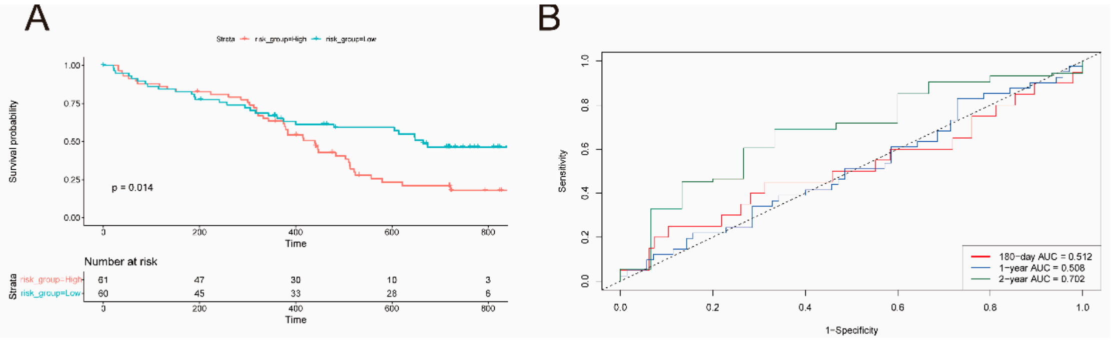

Supplementary Figure S1. Evaluation of the Prognostic Model in the Validation Cohort. **A** Kaplan-Meier survival curves for cervical cancer patients in the validation cohort, stratified into high-risk and low-risk groups based on the median risk score derived from the prognostic model. **B** Time-dependent receiver operating characteristic (ROC) curves of the prognostic model for predicting 0.5-year, 1-year, and 2-year overall survival in the validation cohort.

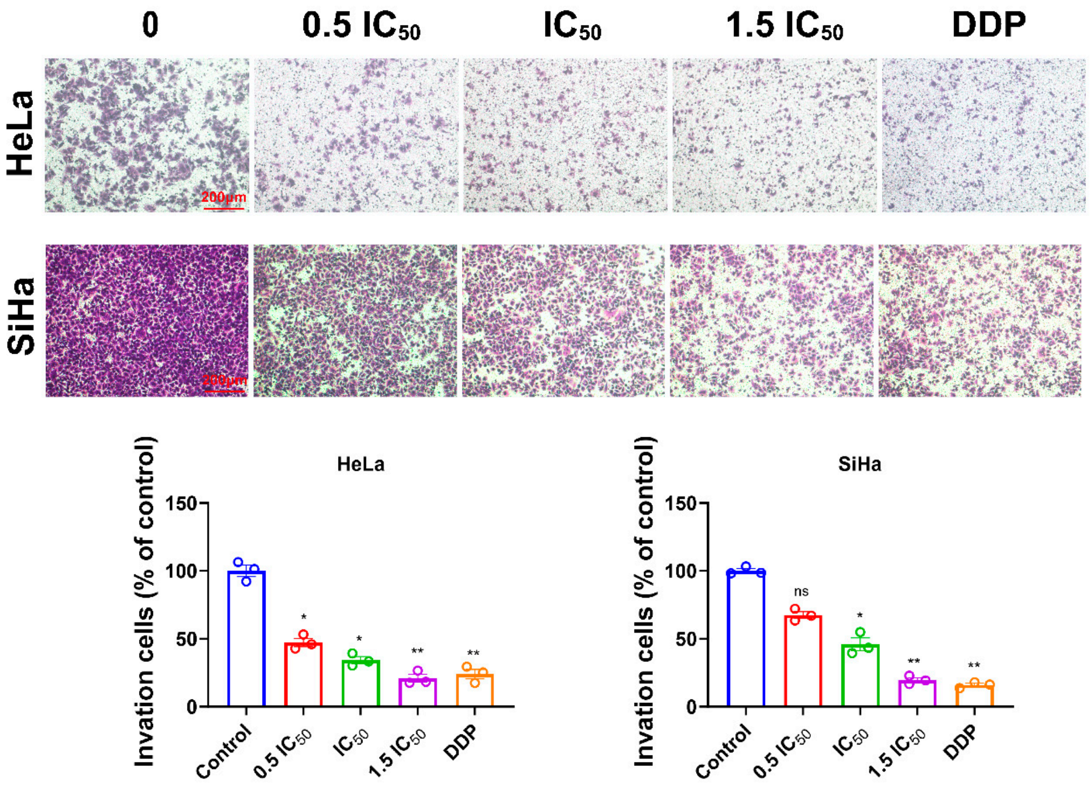

Supplementary Figure S2. XAFHO Suppresses Invasion of Cervical Cancer Cells. A-B rTranswell invasion assays were performed to evaluate the invasive capacity of HeLa and SiHa cells following 24 h treatment with CXHO at indicated concentrations (0.5 IC_{50}, IC_{50}, 1.5 IC_{50}). The upper chambers were pre-coated with Matrigel, and cells that invaded through the Matrigel and polycarbonate membrane were stained and imaged (scale bar: 100 μm). Lower panels: quantitative analysis of invaded cells per field. All data are presented as mean ± SD. *$P<0.05$, **$P<0.01$, ***$P<0.01$vs. control group.

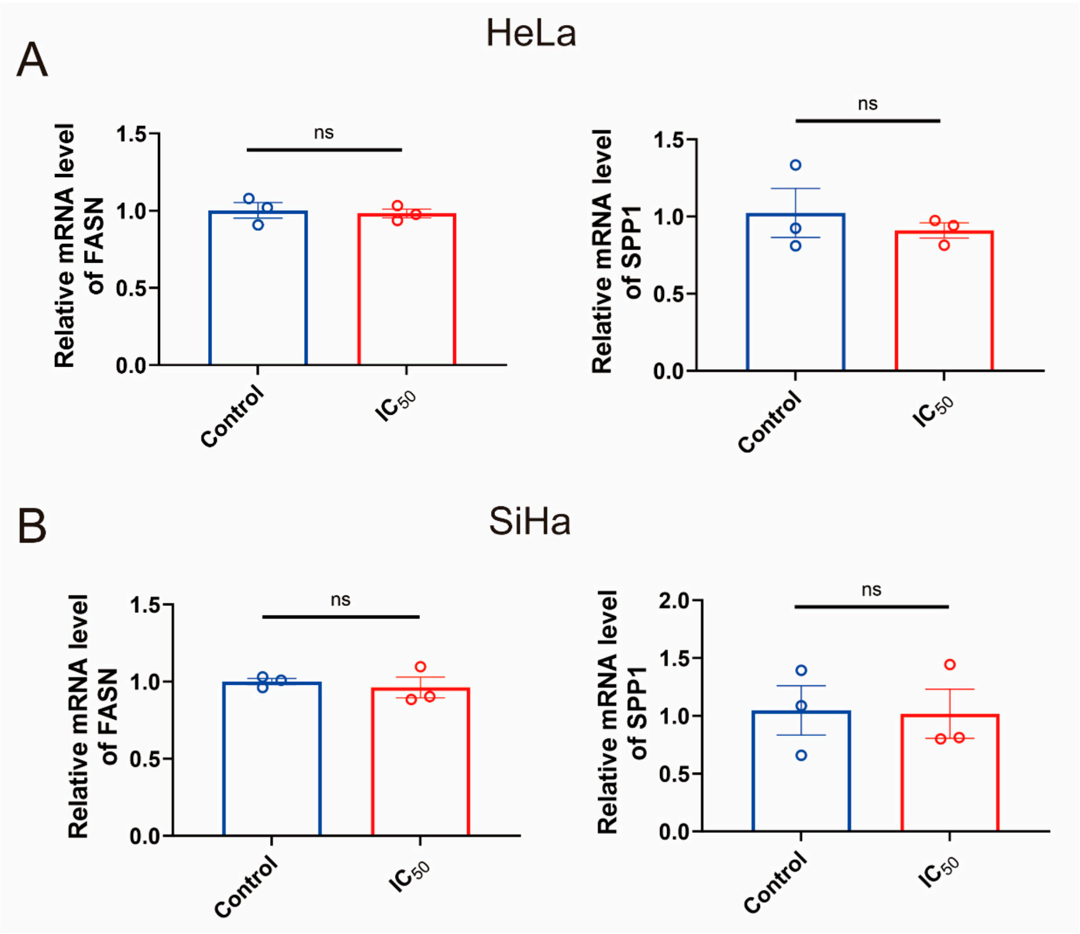

Supplementary Figure S3. Effect of XAFHO on the mRNA expression levels of FASN and SPP1 in cervical cancer cells. A-B Relative mRNA expression levels of FASN and SPP1 in HeLa cells and SiHa treated with XAFHO at the IC_{50} concentration for 24h, as determined by RT-qPCR. Data are presented as mean ± SD.

Table S1. Information on bioactive constituents identified in CXHO.

Herb name	Mol ID	Molecule name	OB(30%)	DL
**Piperis Longi Fructus (Biba)**	MOL001558	sesamin	56.55	0.83
	MOL001559	piperlonguminine	30.71	0.27
	MOL001560	pipernonaline	51.32	0.33
	MOL001561	dehydropipernonaline	47.73	0.24
	MOL001586	N-(2,5-dimethoxyphenyl)-4-methoxybenzamide	60.70	0.18
	MOL001592	piperine	45.52	0.23
	MOL001594	Pisatin	88.05	0.64
	MOL001601	1,2,5,6-tetrahydrotanshinone	38.75	0.36
	MOL001607	ZINC03982454	36.91	0.76

**Rhizoma Alpiniae Officinarum (Gaoliangjiang)**	MOL000358	beta-sitosterol	36.91	0.75
	MOL000359	sitosterol	36.91	0.75
	MOL001771	poriferast-5-en-3beta-ol	36.91	0.75
	MOL002543	(2S,3R)-2-(3,4-dimethoxyphenyl)-5,7-dimethoxychroman-3-ol	51.89	0.37
	MOL002544	1,7-diphenyl-5-hydroxy-3-heptanone	61.90	0.18
	MOL002554	5-methoxy-1,7-diphenyl-3-heptanone	68.29	0.20
	MOL002556	7-Methoxy-8-(2'-ethoxy-3'-hydroxy-3'-methybutyl)coumarin	40.36	0.21
	MOL002563	galangin	45.55	0.21
	MOL002565	Medicarpin	49.22	0.34
	MOL002575	butyl-2-ethylhexyl phthalate	44.52	0.22
	MOL000354	isorhamnetin	49.60	0.31
	MOL000422	kaempferol	41.88	0.24
	MOL000098	quercetin	46.43	0.28
**Piper nigrum L (Hujiao)**	MOL002840	Cryptopimaric acid	39.58	0.28
	MOL002847	Piperolein B	32.26	0.41
	MOL002848	cis-Piplartine	96.65	0.24
	MOL002862	pipercide	42.72	0.43
	MOL002863	(E)-7-(1,3-benzodioxol-5-yl)-1-piperidino-hept-6-en-1-one	54.19	0.31
	MOL002864	(2E,4Z)-5-(1,3-benzodioxol-5-yl)-1-piperidino-penta-2,4-dien-1-one	37.52	0.23
	MOL002866	(2E,4E,8E)-9-(1,3-benzodioxol-5-yl)-N-isobutylnona-2,4,8-trienamide	65.90	0.33
**Zingiber officinale Roseco (Ganjiang)**	MOL002501	[(1S)-3-[(E)-but-2-enyl]-2-methyl-4-oxo-1-cyclopent-2-enyl]	62.52	0.31

		(1R,3R)-3-[(E)-3-methoxy-2-methyl-3-oxoprop-1-enyl]-2,2-dimethylcyclopropane-1-carboxylate		
	MOL002514	Sexangularetin	62.86	0.30

Table S2. Multivariable Cox regression analysis of the 11 prognostic genes in cervical cancer.

Gene	HR	HR.95L	HR.95H	pvalue	Coefficient
**FASN**	1.493328	1.189054	1.875464	0.000562	0.082843
**ACACA**	1.9189	1.267259	2.905623	0.002078	0.236104
**IL12B**	0.795577	0.685152	0.923799	0.002703	-0.11401
**HTR3A**	0.879233	0.79779	0.968989	0.009455	-0.05141
**BCL2**	0.662621	0.5205	0.843548	0.000834	-0.17076
**MMP1**	1.129923	1.042815	1.224308	0.002843	0.109324
**MMP3**	1.129148	1.036484	1.230097	0.005433	0.025755
**SULT1E1**	0.90099	0.827648	0.980831	0.016095	-0.11217
**SPP1**	1.18299	1.048327	1.33495	0.006422	0.028358
**E2F1**	0.670659	0.4886	0.920557	0.013428	-0.05981
**HK2**	1.359549	1.077713	1.715089	0.009559	0.07944

Table S3. The composition of XAFHO.

Chinese name	Plant name/Latin name	Genus Family	Proportion (g)	Part used
Biba	Piper longum L	Piperaceae	200	Fruit
Gaoliangjiang	Alpinia officinarum Hance	Zingiberaceae	200	Rhizome
Hujiao	Piper nigrum L	Piperaceae	200	Fruit
Ganjiang	Zingiber officinale Roscoe	Zingiberaceae	200	Rhizome
Jinqianbaihuashe	Zaocys dhumnades	Colubridae	200	Skin

The plant name has been checked with https://wfoplantlist.org/ and processed honey is used as an excipient (at a dosage of 300g).
